# Plasmid-driven clonal expansion of multidrug-resistant monophasic *Salmonella* Typhimurium in a Global Food Trade Hub

**DOI:** 10.1080/22221751.2025.2542251

**Published:** 2025-08-10

**Authors:** Dingjie Huang, Ziqi Wu, Yixiang Jiang, Lulu Hu, Rui Cai, Xi Yang, Chen Du, Shiting Chen, Panpan Yang, Bingchan Guo, Xiaolu Shi, Shuang Wu, Yinghui Li, Zhemin Zhou, Qinghua Hu

**Affiliations:** a School of Public Health, Shanxi Medical University, Taiyuan, People's Republic of China; b Shenzhen Center for Disease Control and Prevention, Shenzhen, People's Republic of China; c Department of Public Health Laboratory Sciences, School of Public Health, Hengyang Medical School, University of South China, Hengyang, People's Republic of China; d National Key Laboratory of Intelligent Tracking and Forecasting for Infectious Diseases, National Institute for Communicable Disease Control and Prevention, Chinese Center for Disease Control and Prevention, Beijing, People's Republic of China; e Cancer Institute, The Second Affiliated Hospital of Soochow University, Suzhou Medical College, Soochow University, Suzhou, People's Republic of China

**Keywords:** *Salmonella* Typhimurium monophasic variant, multidrug resistance (MDR), plasmid, transmission routes, food handlers

## Abstract

Shenzhen, a major port city with a heavily imported food supply, offers a critical setting to examine the spread and adaptation of multidrug-resistant *Salmonella* 1,4,[5],12:i: – (*S.* 1,4,[5],12:i:-). This study integrates 17 years of genomic, epidemiological, and food safety data. We explored the serovar’s population structure, antibiotic resistance gene (ARG) patterns, and transmission dynamics locally and globally. Our analyses revealed substantial rise in *S*. 1,4,[5],12:i: – prevalence among non-typhoidal *Salmonella* isolates over the past 17 years, from 2.27% in 2007 to 24.79% in 2023. *S*. 1,4,[5],12:i: – was predominated by ST34 (97.9%), with high genotypic resistance to aminoglycosides (100%), tetracyclines (96.6%), β-lactams (89.3%), and sulphonamides (88.5%). Phylogenetic analysis separated *S.* 1,4,[5],12:i: – into four clades. Clade 4, first detected in Shenzhen in 2013, emerged as the predominant lineage by 2023 (56.9%). This clade exhibited minimal genetic diversity (≤ 38 core SNPs), with adaptive traits linked to the acquisition of resistance-associated plasmids. Notably, plasmid-driven ARGs, including carbapenem resistance genes, have emerged as a growing concern. Transmission analysis identified two key transmission dynamics: transient outbreaks primarily involving food handlers and persistent lineages sustained through local and international spread, often facilitated by the food supply chain. These findings underscore the role of occupational carriers and imported food products in the dissemination of ARGs, emphasizing the need for enhanced surveillance and improved health and hygiene practices for food handlers. This study provides a comprehensive molecular epidemiological framework for addressing multidrug-resistant *Salmonella* in globalized urban food hubs, offering a foundation for future surveillance and control efforts.

## Introduction

Nontyphoidal *Salmonella* (NTS) ranks among the most prevalent pathogens responsible for foodborne gastrointestinal infections worldwide, typically causing self-limiting illnesses but occasionally leading to severe outcomes [1]. Globally, NTS is estimated to cause 3.4 million enteric infections and over 680,000 deaths annually [2]. In China, the incidence of NTS infections exceeds 0.6% of the population and continues to rise, posing an increasing public health burden [3].

To date, more than 2,600 *Salmonella* serotypes have been identified based on surface antigen variations, with over 2,000 in subspecies *enterica* [4]. Among these, *Salmonella enterica* serovar Typhimurium stands out as a leading cause of gastroenteritis. This serovar is further subdivided into sequence types (STs), with ST19, ST34, ST36, and ST313 contributing disproportionately to the global disease burden [5,6]. Notably, *Salmonella* 1,4,[5],12:i: – (hereafter *S.* 1,4,[5],12:i:-), a monophasic variant of Typhimurium in ST34, diverged from the ancestral ST19 in the late 1980s and has since become a major global pathogen [7]. The variant has rapidly disseminated across continents, now ranking among the top five *Salmonella* serotypes linked to human and animal infections [8,9]. Recent multinational outbreaks linked to chocolate products underscore its capacity for foodborne transmission and persistence within global supply chains [10,11], emphasizing the need to understand its evolutionary trajectory and resistance mechanisms.

The global spread of *S.* 1,4,[5],12:i: – coincides with alarming trends in antimicrobial resistance (AMR), largely driven by antibiotic misuse in livestock production and clinical settings [12]. This variant exhibits higher resistance rates than most NTS serotypes, particularly to first-line antibiotics such as ampicillin, streptomycin, sulphonamides, and tetracyclines [13–15]. Over 65% of *S.* 1,4,[5],12:i: – isolates in public databases are multidrug-resistant (MDR), with Chinese strains showing even higher prevalence (86%) [16–18]. Critical ARGs, including *qnrS1* (quinolones), *bla*_CTX-M-55_ (third-generation cephalosporins), *mph*(A) (macrolides), and *mcr*-3 (colistin), further compromise therapeutic options [7]. The recent study in Guangdong reported that the *S.* 1,4,[5],12:i: – exhibits high resistance to first-line antimicrobials, with nearly 50% of clinical isolates showing resistance to fluoroquinolones and cephalosporins. It not only restricts the available treatment options for drug-resistant infections but may also lead to prolonged illness, increased risk of complications, and heavier burdens on public health. The associated rise in treatment costs can aggravate the economic burden on affected households. Thus, strengthening measures to prevent the spread of antibiotic resistance is of critical importance.

Horizontal gene transfer mediated by mobile genetic elements (MGEs), particularly plasmids, plays a pivotal role in disseminating these resistance determinants [19]. IncHI2/2A and IncA/C2 plasmids are frequently implicated [20], with studies demonstrating their low fitness costs in *S.* 1,4,[5],12:i: – strains and their association with resistance gene clusters in Southeast Asia [21]. Such plasmid-driven resistance threatens to erode the efficacy of critically important antimicrobials (CIAs), necessitating genomic surveillance to track emerging threats.

Despite the recognized global significance of *S*. 1,4,[5],12:i:-, most previous studies have been limited by short surveillance periods, single-source sampling, or restricted geographic scope, hampering our understanding of long-term evolutionary dynamics and transmission patterns [22]. Effective surveillance of foodborne pathogens requires integrated multi-source approaches that bridge clinical, food safety, and occupational health data to capture the complex transmission networks characteristic of One Health pathogens [23].

Particularly, despite growing AMR concerns in China, the epidemiology and evolutionary dynamics of *S.* 1,4,[5],12:i: – remain largely uncharacterized [24]. Shenzhen, as a major port city, exemplifies this risk due to its reliance on external food supplies. Limited local agricultural production and high reliance on imported food not only complicate pollution traceability but also increase regulatory challenges due to cross-regional food distribution. Inadequacies in cold chain transport or quarantine procedures further facilitate the introduction of MDR strains. Food handlers, a critical nexus in transmission chains, may inadvertently facilitate outbreaks through contaminated products, a risk compounded by gaps in health surveillance [25].

To address these knowledge gaps and methodological limitations, this study represents a comprehensive, multi-source genomic surveillance analysis of *S*. 1,4,[5],12:i: – integrating clinical, food safety, and occupational health data over an extended 17-year period (2007–2023). Our approach leveraged three complementary surveillance programmes in Shenzhen: infectious diarrhoea surveillance, food safety monitoring, and occupational health screening, providing unprecedented insight into transmission dynamics across different sectors. By analysing 1,908 *S.* 1,4,[5],12:i: – isolates alongside global genomes, we elucidate the serovar’s population structure, resistance gene distribution, and transmission routes, providing a foundation for targeted interventions in high-risk urban settings. This multi-source, longitudinal approach enables identification of sector-specific transmission patterns, evolutionary hotspots, and resistance emergence in an unprecedented resolution, representing a paradigm shift toward comprehensive One Health genomic epidemiology.

## Materials and methods

### Strain collection and ethics

*S.* 1,4,[5],12:i:- isolates were collected through Shenzhen’s Infectious Diarrhea Disease Surveillance (IDDS), Food Safety Surveillance (FSS), and Occupational Food Handler Screening (OFHS) between 2007 and 2023. The IDDS isolates were passively collected from diarrhoeal outpatients at 16 sentinel hospitals. The inclusion criteria for IDDS required: (1) ≥ 3 bowel movements per day; (2) altered stool consistency, including loose stools, watery stools, mucous stools, or bloody stools; and (3) patients who had not used antibiotics prior to consultation. The FSS isolates were obtained through active surveillance from food samples collected monthly in supermarkets, wet markets, catering facilities, and street retailers across all administrative districts of Shenzhen. Stratified random sampling was conducted by food category, manufacturer, and production batch to ensure comprehensive coverage. The OFHS isolates were derived from rectal swabs collected during routine health examinations of food handlers in Longgang and Longhua districts. These individuals were occupationally engaged in handling ready-to-eat foods. Positive detection of *Salmonella* in faecal specimens (rectal swabs) indicated asymptomatic carriage, with data collected through passive surveillance. No statistical methods were used to predetermine sample size, and all available isolates meeting inclusion criteria were retained for analysis. The study did not involve randomization, blinding, or direct patient participation. Informed consent was waived as data were obtained through routine public health surveillance protocols. All personal identifiers were anonymized prior to analysis. Ethics approval was obtained from the Shenzhen Centers for Disease Control and Prevention Clinical Research Ethics Committee (SZCDC-IRB2025058).

### Epidemiological data integration

We also collected the source, geographic origin, and time of isolation for all isolates from Shenzhen’s IDDS, FSS, and OFHS programmes. Geographic and temporal metadata were used to trace the movement of strains within Shenzhen and the broader Guangdong region. Isolation source was classified into human, animal, food, and environmental categories, and correlations between transmission clusters and outbreak data were assessed.

### Whole genome sequencing (WGS)

All genomic DNAs were extracted using the Baypure Magnetic Bead Bacterial DNA Extraction Kit (Dalian, China). Libraries were prepared with the NEBNext Ultra DNA Library Prep Kit (NEB, USA) with an average insert size of 350 bp and sequenced on the Illumina NovaSeq 6000 platform (Novogene, Tianjin, China) to generate paired-end 150 bp reads. Each isolate achieved a minimum theoretical coverage of 100×, yielding approximately 1 GB of raw data per genome. Sequencing data are deposited in the NCBI Sequence Read Archive (BioProject ID: PRJNA1226905).

### Bioinformatics workflow

Raw reads were quality-trimmed using Trimmomatic v0.39 [26] and assembled de novo with SPAdes v3.9.1 [27] via the Shovill v1.1.0 pipeline (k-mer range: 21–127). The assembly parameters used were the default parameters for paired – end sequencing. Assembly quality was assessed using QUAST v5.0.2 [28]. According to the White-Kauffmann – Le Minor scheme, *Salmonella* Typhimurium and its monophasic variants are serotyped using the slide agglutination method with SSI® *Salmonella* diagnostic antisera (Statens Serum Institut). Serotyping and sequence typing (MLST) were confirmed using SISTR v1.1.1 [29] and the PubMLST scheme (MLST v2.0), respectively. ARGs and chromosomal mutations were identified with ResFinder v4.3.3 [30,31], while plasmid replicons were predicted using PlasmidFinder v2.1 [32]. PlasmidFinder was applied to predict plasmid replicons with sequence identity ≥ 95% and coverage ≥ 90%. The co-linearity relationship between ARGs and plasmids was predicted using Klety [33]. ABRicate v0.8.10 (https://github.com/ tseemann/abricate) workflow to screen for virulence factors by comparing against the VFDB [34] database. *Salmonella* pathogenicity islands (SPIs) were detected using SPIFinder v2.0 with default settings of identity ≥ 95% and coverage ≥ 90%. Core genome SNPs were called against the reference strain *S*. Typhimurium LT2 (GenBank: NC_003197) [35] via Snippy v4.3.6 [36], with recombination regions removed using Gubbins v2.4.1 [37]. A maximum-likelihood phylogenetic tree was constructed from the SNP matrix using FastTree v2.1.10 [38], visualized with iTOL [39] and GrapeTree v1.5.0 [40]. Temporal evolutionary analysis was performed with TreeTime v0.11.4 [41]. Transmission clusters were defined as isolates sharing ≤ 3 pairwise SNPs [42].

### Antimicrobial susceptibility testing

Based on the following criteria, 256 bacterial isolates in this study were selected for AST (Antimicrobial Susceptibility Testing). According to the resistance phenotype patterns predicted by ResFinder for 18 antibiotics, five isolates were randomly chosen for each resistance phenotype pattern between 2007 and 2023, with all isolates included if fewer than five were available.

The microbroth dilution method was used to evaluate strains for susceptibility testing. The BD PhoenixTM M50 automated microbiology system and customized Gram-negative panels (RUONMIC-801) were purchased from BD (USA). Results were interpreted according to CLSI M100-S31 criteria, with *Escherichia coli* ATCC 25922 as the quality control strain. The minimum inhibitory concentrations (MICs) of 18 antimicrobial agents across 9 classes were determined: amikacin (AMI), ampicillin (AMP), ampicillin/sulbactam (AMS), aztreonam (AZT), cefotaxime (CTX), cefoxitin (FOX), ceftazidime (CAZ), ceftazidime/avibactam (CZA), colistin (CT), ertapenem (ETP), imipenem (IPM), meropenem (MEM), ciprofloxacin (CIP), nitrofurantoin (NFT), tetracycline (TET), tigecycline (TIG), chloramphenicol (CHL), and trimethoprim/sulfamethoxazole (SXT). Susceptibility results were categorized as susceptible (S), intermediate (I), or resistant (R).

### Phylogenetic and epidemiological context

A global phylogenetic framework was constructed by integrating 1908 Shenzhen isolates with 1,523 publicly available *S.* 1,4,[5],12:i:- genomes from EnteroBase and NCBI. This dataset included 382 isolates from 11 Guangdong cities (Dongguan, Foshan, Guangzhou, Heyuan, Jiangmen, Jieyang, Maoming, Shaoguan, Yangjiang, Zhongshan, Zhuhai), 187 isolates from 20 other Chinese provinces, and 954 isolates from 18 countries spanning 1949 to 2023. The combined collection comprised 3,431 genomes, predominantly from humans (2,749; 80.1%), with additional isolates from animals (530; 15.4%), food (134; 3.9%), and environmental sources (38; 1.1%).

### Statistical analysis

Statistical analysis and figures were performed using Python and R, and the heatmap was created using the Biozeron Cloud Platform (http://www.cloud.biomicroclass.com/CloudPlatform) [43]. The correlation coefficients and significance levels (*P*-values) between ARGs and plasmids were calculated using the Spearman method. The results were classified into three categories based on the magnitude of the correlation coefficients: strong correlation (|*r*| > 0.7), moderate correlation (0.3 < |*r*| ≤ 0.7), and weak correlation (|*r*| ≤ 0.3).

## Results

### Strain characteristics and epidemiological trends

**Figure 1.**
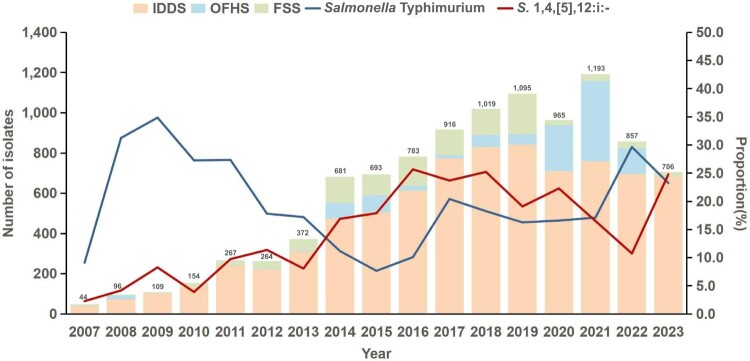
The detection situation of non-typhoidal *Salmonella* in Shenzhen from 2007 to 2023. The bar charts indicate the total counts of *Salmonella* and the total counts of non-typhoidal *Salmonella* from different sources. The top of the bar chart indicates the total NTS isolates per year. The line charts depict the proportions of *Salmonella* Typhimurium and *S*.1,4,[5],12:i:- among non-typhoidal *Salmonella*. (Abbreviations: IDDS = Infectious Diarrhoea Disease Surveillance; FSS = Food Safety Surveillance; OFHS = Occupational Food Handler Screening.).

Between 2007 and 2023, a total of 10,214 non-typhoidal *Salmonella* (NTS) isolates were collected in Shenzhen, of which 1,908 (18.7%) were identified as the monophasic variant *S.* 1,4,[5],12:i:-. The proportion of this variant among NTS isolates rose from 2.27% in 2007 to 24.79% in 2023 (Figure 1). Geographically, isolates were predominantly clustered in Longgang (548/1,907, 28.8%) and Bao’an (451/1,907, 23.6%) districts (Supplementary Figure 1).

The majority of the human samples (1,740, 94.6%) were from diarrhoeal patients through sentinel surveillance, supplemented by 100 isolates (5.4%) from food handlers screened during occupational health exams. The majority of the patients were male (938/1,661, 56.5%) and under age three (1,115/1,643, 67.9%). Clinical manifestations included diarrhoea (97.4%), fever (35.8%), abdominal pain (17.9%), and vomiting (8.6%) (Table 1). Additionally, food safety surveillance identified 68 *S.* 1,4,[5],12:i: – isolates, with pork liver and pork meat each accounting for 30.9% (21/68) of contaminated products.

**Table 1.** Demographic and clinical symptom characteristics of S. 1,4, [5], 12:i:- infections in Shenzhen, China, 2007–2023.

	Clade1	Clade2	Clade3	Clade4
**Sources**	30	395	619	864
OFHS	1 (3.33%)	16 (4.05%)	32 (5.17%)	51 (5.90%)
IDDS	21 (70%)	360 (91.14%)	567 (91.60%)	792 (91.67%)
FSS	8 (26.67%)	19 (4.81%)	20 (3.23%)	21 (2.43%)
**Sex**	22	356	572	806
Female	5 (22.73%)	159 (44.66%)	253 (44.23%)	354 (43.92%)
Male	17 (77.27%)	197 (55.34%)	319 (55.77%)	452 (56.08%)
**Age (year)**	21	350	568	799
0–3	15 (71.44%)	229 (65.42%)	362 (63.73%)	509 (63.70%)
3–6	1 (4.76%)	19 (5.43%)	37 (6.51%)	54 (6.76%)
6–10	–	10 (2.86%)	9 (1.58%)	7 (0.88%)
10–20	–	5 (1.43%)	19 (3.35%)	27 (3.38%)
20–30	2 (9.52%)	33 (9.43%)	56 (9.86%)	66 (8.26%)
30–40	1 (4.76%)	23 (6.57%)	34 (5.99%)	50 (6.26%)
40–50	1 (4.76%)	11 (3.14%)	23 (4.05%)	39 (4.88%)
50–60	–	7 (2%)	13 (2.29%)	31 (3.88%)
60–70	–	3 (0.86%)	9 (1.58%)	8 (1%)
70–80	1 (4.76%)	5 (1.43%)	3 (0.53%)	4 (0.50%)
80–90	–	3 (0.86%)	3 (0.53%)	4 (0.50%)
90–100	–	2 (0.57%)	–	–
**Symptom**	19	289	470	658
Diarrhoea	18 (94.74%)	284 (98.27%)	457 (97.23%)	639 (97.11%)
Abdominal pain	3 (15.79%)	59 (20.42%)	88 (18.72%)	107 (16.26%)
Vomiting	2 (10.53%)	29 (10%)	42 (8.94%)	50 (7.60%)
Fever	7 (36.84%)	116 (40.14%)	175 (37.23%)	216 (32.83%)

### Population structure and evolutionary dynamics

**Figure 2.**
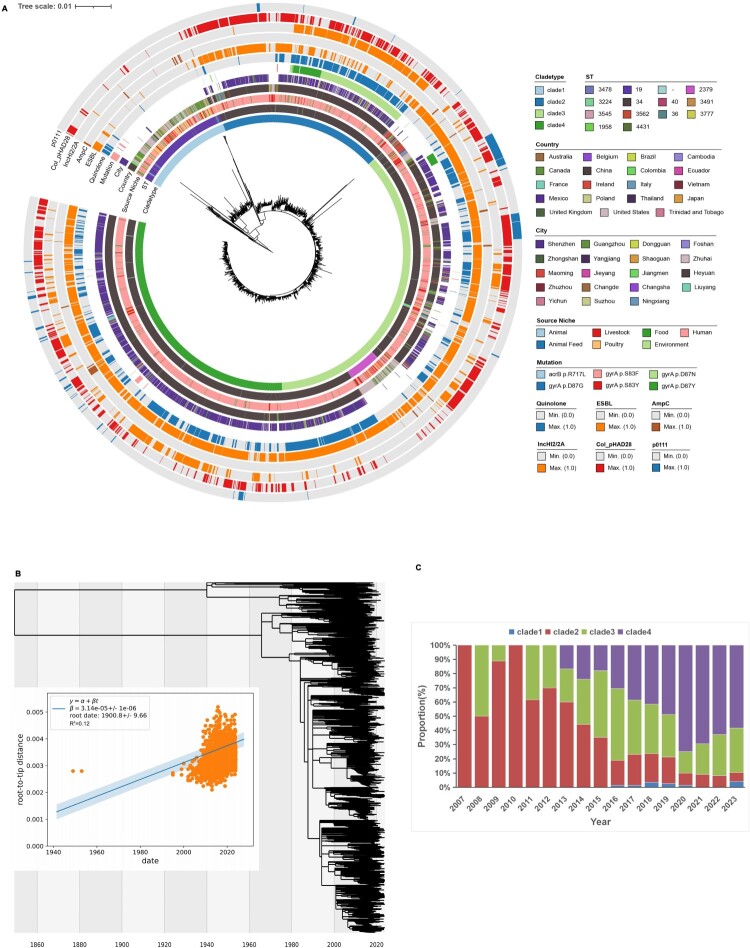
Population structure and epidemiology of *S.* 1,4,[5],12:i:- strains. (A) Population structure of *S*. 1,4,[5],12:i:-. Maximum-likelihood phylogenetic tree constructed based on the 1908 genome sequences reported in this study and 1,523 publicly available *S.* 1,4,[5],12:i:- strains with genotypic information and metadata. The scale bar indicates the number of substitutions per site per genome. Metadata is visualized on the concentric rings according to the legend, from inside to outside;1. Clade branch, 2. Sequence type (ST), 3. Source of isolates, 4–5. Country and City, 6–9. Presence of multidrug resistance genes markers (QRDR mutation, quinolone, ESBL, and *AmpC*), 10–12. Presence of plasmid replicons (IncHI2/2A, Col_pHAD28, p0111). (B) The time-scaled phylogeny of *S*. 1,4,[5],12:i:- strains, derived from time divergence analysis conducted with TreeTime. The illustration depicts the phylogenetic estimation of the evolutionary rate of isolates using TreeTime on a time-scaled phylogeny. The X-axis represents time, and the Y-axis indicates root-to-tip regression. (C) Epidemiological prevalence of clade branches in Shenzhen. The X-axis represents time, and the Y-axis shows the stacked percentage.

Multilocus sequence typing (MLST) classified the 1,908 Shenzhen isolates into five sequence types (STs): ST34 (1,868/1,908, 97.9%), ST19 (23/1,908, 1.2%), ST36, ST1958, and ST2379, plus eight untypable isolates. A maximum-likelihood phylogeny integrating 1,908 Shenzhen isolates with 1,523 global genomes resolved four major clades (Figure 2(a)): Clade 1 comprised both ST19 and ST36 (Shenzhen ST36 isolates were all isolated in 2023), while Clades 2–4 were predominantly ST34 (> 99.3% in each).

Genetic diversity varied significantly across clades. Clade 1 exhibited the highest SNP divergence (≤ 467 SNPs), followed by Clades 2 (≤ 77 SNPs), Clades 3 (≤ 85 SNPs), and Clades 4 (≤ 49 SNPs). Shenzhen isolates within Clade 4 showed even lower genetic diversity (≤ 38 SNPs), suggesting localized clonal expansion. Temporally, ST34 dominated throughout the study period (2007–2023), with clade compositions vary with time: Clade 2 emerged in 2007 and declined after 2012, followed by Clade 3 which peaked in 2016, descreased afterward and reemerged after 2020. Notably, Clade 4 rose rapidly after its 2013 introduction, becoming the predominant lineage by 2020 (56.9% in 2023; Figure 2(c)).

Root-top-tip analysis revealed median-level, yet significant molecular clock signal (*R^2^* = 0.12, *p* < 0.001), which is common in large-scale analysis [44]. Time-scaled phylogeny estimated a substitution rate of 1.0 × 10^−7^ substitutions per site per year (95% *HPD*: 9.5 × 10^−8^–1.04 × 10^−7^), with the most recent common ancestor (MRCA) dated to approximately 1900 (*CI* 95%: 1891–1910). This estimate was in line with previous estimates for ST34 *S*. 1,4,[5],12:i:- in Guangdong Province [24].

We observed genetically closely related pairs between Shenzhen isolates and those from Canada, Ireland, the United States, and Southeast Asia (Vietnam, Japan, Thailand), indicating recent international introductions. Notably, Canadian isolates frequently clustering within 0–7 SNPs of Shenzhen strains, likely representing travel-related transmissions. Domestic transmission routes connected Shenzhen to Shanghai, Guangxi, Chongqing, and Hunan (0–10 SNPs), while intra-provincial spread centred on the Pearl River Delta (Guangzhou, Zhuhai, Dongguan, Foshan, Zhongshan), Yangjiang, Heyuan, and Shaoguan (0–6 SNPs), indicating sustained local reservoirs.

### Molecular epidemiology of transmission clusters

**Figure 3.**
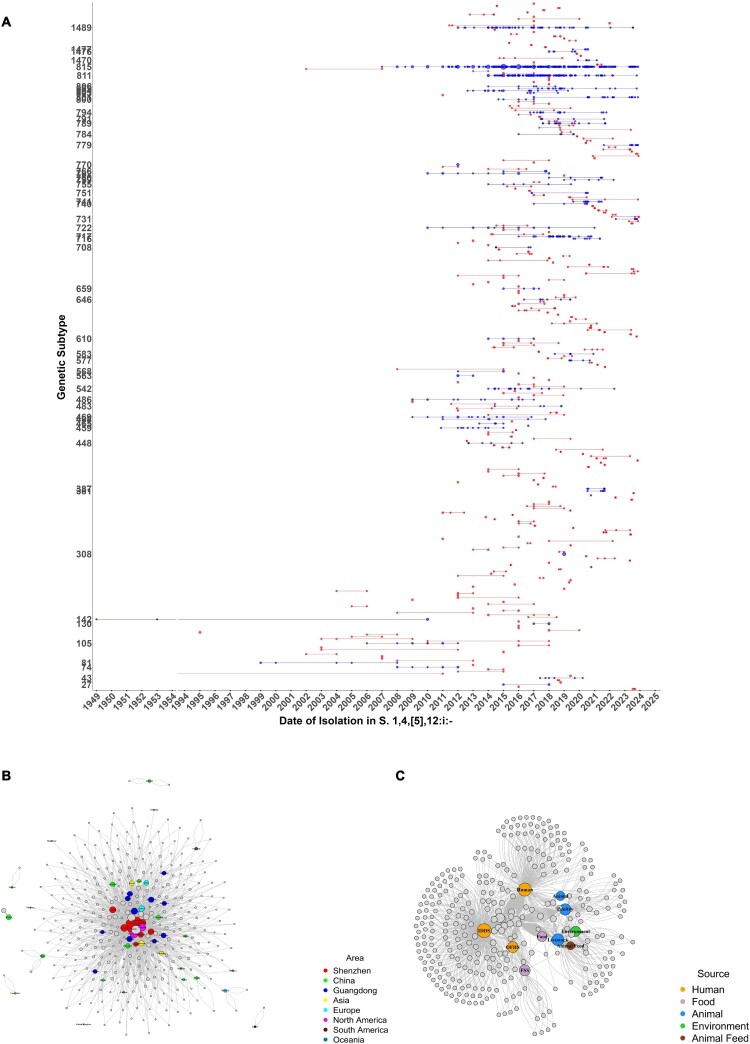
Strains were grouped into clusters based on an SNP threshold of ≤3. Only clusters containing more than two strains are shown. (A) Timing and Size of *S*. 1,4,[5],12:i:- Genetic Clusters. Clusters are plotted on separate horizontal lines according to the date of isolation. The size of each point represents the relative frequency of strains within the cluster. Blue points indicate clusters with a frequency of ≥ 5 strains, while red points represent clusters with a frequency of < 5 strains. (B) Geographic Dissemination of *S*. 1,4,[5],12:i:- Clusters. The grey nodes represent Clusters, and the colours in the legend indicate clusters from the corresponding regions as shown in the legend. Among them, yellow represents Asia excluding China, green represents China excluding Guangdong Province, and blue represents Guangdong Province excluding Shenzhen City. (C) Transmission Dynamics and Demographics of *S*. 1,4,[5],12:i:- Genetic Clusters. The yellow “Human” includes isolates from other regions and those from Shenzhen IDDS and OHFS sources, while the purple “Food” includes foodborne isolates from other regions and those from Shenzhen FSS sources.

Taking our traditionally applied thresholds of < = 3 SNPs [45–48] (see discussion), we identified 316 transmission clusters, encompassing 58.6% (2,011/3,431) of all genomes, with cluster sizes ranging from 2 to 598 isolates. The majority (50.6%, 160/316) of the clusters persisted for < 1 year, representing transient outbreaks (median duration: 6 months). Some clusters persisted much longer, with a few lasted over decades. Notably, two clusters (Cluster 59 and 142) in the United States had genetically almost identical isolates separated by 42 and 61 years, respectively, suggesting long-term environmental or host adaptation (Figure 3(a)).

Our analysis revealed strong region specificity in the 202 Shenzhen-associated clusters, with 80.8% (968/1,198) of cases concentrated in five districts of Longgang, Bao’an, Futian, Longhua, and Guangming. Geospatial analysis further revealed that many Shenzhen isolates were genomically linked to the isolates across the Pearl River Delta region, including nine clusters involving strains from Guangzhou. One of these clusters involved porcine-derived strains and human diarrhoeal cases. Three clusters were traced to Jieyang, including two linked to contaminated pork liver. (Figure 3). Additionally six clusters linking to Canada, representing international transmission routes.

We found 18 clusters that directly link food handlers to patients, underscoring their key role in foodborne outbreaks. Of particular importance, four clusters simultaneously contained isolates from food, food handlers, and patients. For instance, in cluster 486, a food handler isolate (July 2015) differed by 0 SNP from a pork-derived food sample (April 2016) and a patient isolate from Jieyang (2017), suggesting a plausible “food handler–food–patient” transmission.

### Increasing level of antimicrobial resistance

The antimicrobial susceptibility test results showed that the ARGs detected in 256 strains of *S*. 1,4,[5],12:i:- from Shenzhen exhibited high consistencies with the phenotypic test results (79.7%–100%; Table 2). Notably, we obtained 100% consistencies for AMI (*aac*(6’)-Iaa), TIG (*tet*(X4)), and CT (*mcr*-1.1), and 99.6% consistencies for FOX (predominantly *bla*_CMY-2_). Over 98% consistencies were also obtained for IPM and MEM (*bla*_NDM-5_, 98.8%) and TET (*tet*(B), 98.4%).

**Table 2.** Drug-resistant phenotypes of S. 1,4,[5],12:i:- strains (*n* = 256).

Antibacterial drug types	Antimicrobial agent	Resistance phenotype	No. of isolates	Prediction R/S	No. of consistent	Accordance rate (%)
Aminoglycoside	AMI	R	256	256/0	256	100
	AMP	R	214	212/2	252	98.44
		S	42	2/40		
	ATM	I	18	11/7	225	87.89
		R	78	73/5		
		S	160	19/141		
	CTX	I	1	1/0	243	94.92
		R	104	102/2		
		S	151	11/140		
	FOX	I	6	5/1	255	99.61
Beta-lactamase		R	16	16/0		
		S	234	0/234		
	CAZ	I	3	3/0	204	79.69
		R	61	60/1		
		S	192	51/141		
	ETP	I	6	0/6	249	97.27
		R	2	2/0		
		S	248	1/247		
	IPM	I	1	0/1	253	98.83
		R	3	2/1		
		S	252	1/251		
	MEM	I	0	0	253	98.83
		R	2	2/0		
		S	254	1/253		
Phenicol	CHL	I	3	3/0	237	92.58
		R	130	124/6		
		S	123	13/110		
Colistin	CT	R	13	13/0	255	99.61
		S	243	1/242		
Quinolone	CIP	I	124	94/0	211	82.42
		R	33	33/0		
		S	99	15/84		
Tetracycline	TET	R	219	218/1	252	98.44
		S	37	3/34		
	TIG	R	1	1/0	256	100
		S	255	0/255		
Sulphonamides	SXT	R	88	86/2	222	86.72
		S	168	32/136		

**Figure 4.**
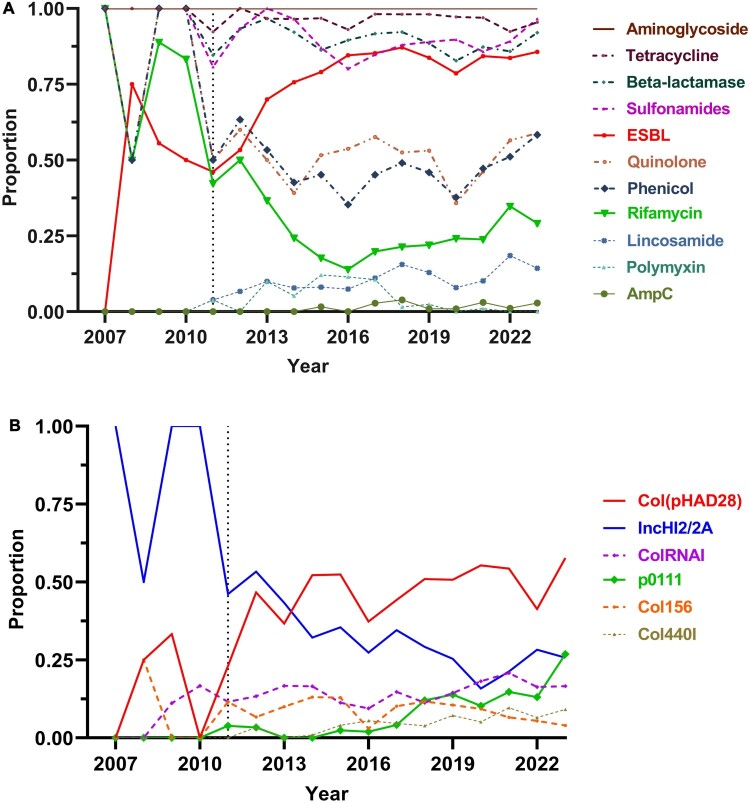
ARGs-related antibiotic types and plasmid replicon carriage associated with *S.* 1,4,[5],12:i:- in Shenzhen. The dashed line on the Y-axis indicates the point from 2011 where the number of *S.* 1,4,[5],12:i:- isolates identified in Shenzhen exceeded 20 strains.(A) Prevalence of main ARGs related to antibiotics among *S.* 1,4,[5],12:i:- in Shenzhen.(B) Prevalence of main plasmid replicons among *S.* 1,4,[5],12:i:- in Shenzhen.

Due to the strong correlation between AMR genotypes and phenotypes in *Salmonella* [49], we used AMR genotyping data to track the evolution of antimicrobial resistance in Shenzhen isolates. High prevalencies were found for resistance genes to aminoglycosides (100%), tetracyclines (96.6%), β-lactams (89.3%), and sulphonamides (88.5%). Genes or mutations conferring resistance to quinolones, chloramphenicol, and colistin were also found in 51.1%, 46.4%, and 4.3% of isolates (Figure 4(a)). Clades 2–4 maintained persistently high carriage rates for aminoglycoside, β-lactam, tetracycline, and sulphonamide resistance genes.

**Figure 5.**
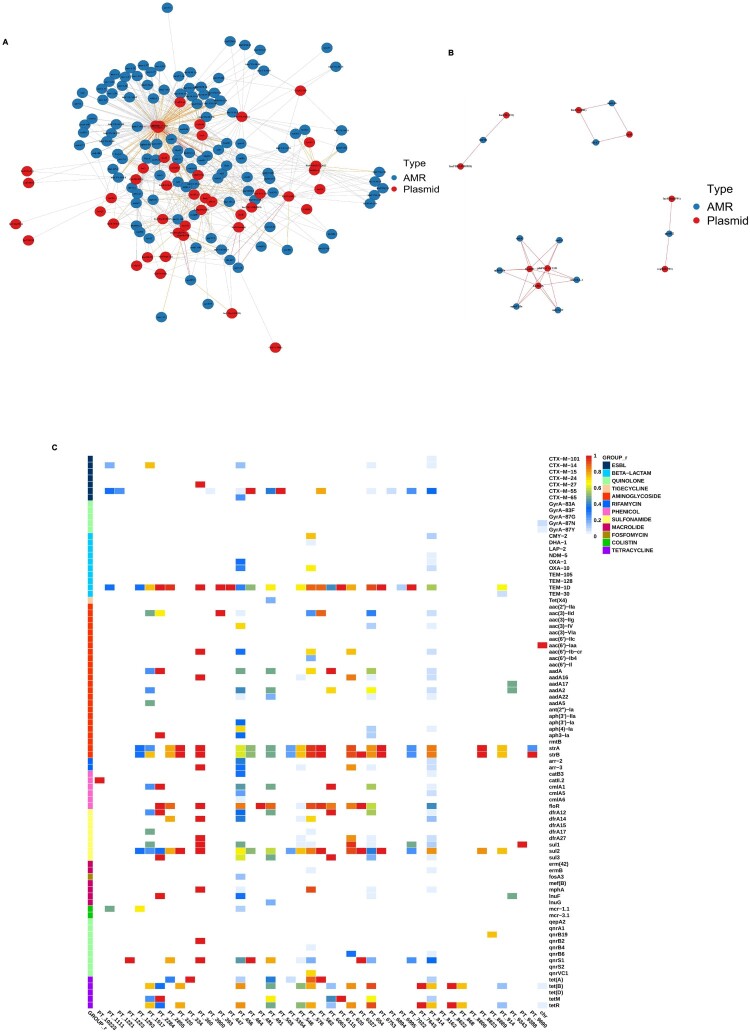
Analysis of the association and co-linearity between resistance genes and plasmids. (A,B) Network diagrams showing the association between resistance genes and plasmids in *S.* 1,4,[5],12:i:- isolates from Shenzhen (*ρ* > 0, *p* < 0.05). Blue dots represent resistance genes, and red dots represent plasmid replicons. Red lines indicate strong associations (*ρ* > 0.7), orange lines indicate moderate associations (0.3 < *ρ* ≤ 0.7), and grey lines indicate weak associations (*ρ* ≤ 0.3). Panel A presents the overall association network between resistance genes and plasmids. Panel B presents the strong association network between resistance genes and plasmids. (C) Co-occurrence relationships between resistance genes and plasmids in *S.* 1,4,[5],12:i:- from Shenzhen analysed using the Kleit method. Gradient colours are used to represent the proportion, with the X-axis indicating plasmids and chromosomes (chr), the Y-axis on the left side indicating types of antibiotics, and the labels on the right side corresponding to the resistance genes.

Notably, Clade 2 exhibited declining resistance genes to chloramphenicol (*floR*), quinolone (*qnrS1*), and rifamycin (*arr*-3) after 2010, aligning with its epidemiological decline. In contrast, Clade 4 demonstrated rising chloramphenicol and quinolone resistance gene rates from 2013 onward, mirroring its dominance after 2020. Clade 3 showed a gradual reduction in colistin resistance genes (*mcr*-1.1) following China’s 2017 ban on colistin as a growth promoter [50], suggesting adaptive selection against this trait (Figure 5(c)).

Aminoglycoside resistance primarily medicated by the genes *aac*(6’)-Iaa, *aph*(3'’)-Ib, and *aph*(6)-Id, with Clade 1 uniquely carrying *aac*(3)-IV, *aadA*1/2, and *aph*(4)-Ia. Tetracycline resistance was predominantly driven by *tet*(B) (declining from 92% to 76%), while the prevalence of *tet*(A) has been increasing from 8% to 24%, displacing *tet*(M) in Clade 1. β-lactam resistance was largely attributed to *bla*_TEM-1B_ (61.1%) and *bla*_OXA-1_ (28.4%), while extended-spectrum β-lactamase (ESBL) genes *bla*_CTX-M-14_ (21.9%) and *bla*_CTX-M-55_ (20.2%) were detected in 352 isolates. Notably, three carbapenem-resistant isolates carrying *bla*_NDM-5_ were detected in 2023, signalling the emergence of resistance to last-line therapies and underscoring a critical public health concern.

Quinolone resistance was primarily driven by the plasmid-mediated gene *qnrS1*, identified in 604 isolates, and chromosomal mutations in *gyrA*, which were present in 15.2% of cases. The most prevalent *gyrA* mutations were D87N (57.4%) and D87Y (31.1%). Clade-specific trends were evident: Clade 1 predominantly featured the *gyrA* S83F mutation (60%), while Clade 2 exhibited a shift from D87N dominance before 2022 to D87Y thereafter. Sulphonamide resistance was overwhelmingly mediated by *sul2* (88.5%). In contrast, resistance genes to macrolides (*mph*(A)), colistin (*mcr*-1.1), and lincosamides (*lnu*(F)) displayed distinct, clade-restricted distributions, highlighting the genetic diversity and adaptability of resistance genes mechanisms across different lineages.

### Plasmids as the main vector of AMR genes

Plasmid dynamics exhibited distinct clade-specific evolutionary trajectories (Figure 4(b)). In Clade 1, ST19 strains predominantly carried Col(pHAD28), IncY, IncFIC(FII), and IncFIB(AP001918), each present in over 50% of isolates, while ST36 strains harboured IncQ1 and IncN.

Among the dominant ST34 lineages in Shenzhen, Clade 2 showed a marked decline in IncHI2/2A and pKPC-CAV1321 plasmid prevalence from 85% in 2010–27% in 2023, with these plasmids concentrated in subclade 2.1 (99.5%). Concurrently, Col(pHAD28) prevalence rose steadily after 2010 before stabilizing at ∼70% post-2012. Col156 peaked in 2020 (63.2%) but subsequently declined to 27.3% in 2023, predominantly in subclade 2.2 (94.7%).

Clade 3 was characterized by the rapid expansion of p0111, which surged from 12% in 2016 to 60% in 2023, often co-occurring with Col(pHAD28) in subclades 3.1 (54.0%) and 3.2 (45.2%). Clade 4, the predominant lineage since 2020, was dominated by Col(pHAD28) (56.9% in 2023), alongside a rising prevalence of ColRNAI (23.5%).

Spearman correlation analysis revealed strong associations between plasmid types and resistance genes (Figure 5(a,b)). IncHI2/2A and pKPC-CAV1321 correlated strongly (*ρ* > 0.7, *p* < 0.05) with *aac*(3)-IV, *aadA1*, *aph*(3’)-Ia, *aph*(4)-Ia, *bla*_OXA-1_, and *cmlA1*, while IncFII(pKP91) and repB(R1701) exhibited perfect co-occurrence with *qnrB52* (ρ = 1). IncFIA(HI1) and IncR plasmids showed robust linkages to *aad*A16 and *dfr*A27 (*ρ* > 0.8), and IncFIC(FII)/IncFIB(AP001918) strongly associated with *tet*(M) (*ρ* > 0.7). Plasmid p0111 displayed moderate correlations with *dfrA14* and *tet*(A) (*ρ* > 0.3).

KleTy-based analysis further resolved plasmid-type (PT) specificity (Figure 5(c)). PT_456 emerged as a multidrug-resistant hub, carrying aminoglycoside (*aac*(3)-IId/IV, *aac*(6’)-Ib-cr, *aad*A/A2, *aph*(3’)-Ia/4-Ia), ESBL (*bla*_CTX-M-14/65_), colistin (*mcr*-1.1), and sulphonamide (*sul1*/3) genes. PT_6753 universally harboured *sul2* (100%) and *tet*(B), alongside *bla*_TEM-1D_ (99.4%), while PT_464 carried *bla*_CTX-M-55_ and *qnrS1*.

PT_7643 was nearly exclusive for *tet*(B) (99.8%), and PT_8823 universally carried *strAB* (100%). Other PTs exhibited niche roles: PT_320 carried *strAB*, *tet*(A), and *sul2*; PT_654 combined *tet*(B), *bla*_TEM-1D_, and *str*AB; PT_620 linked *strAB* to *sul1*/*sul2*; and PT_6063 paired *cmlA1* with *aadA*. These findings underscore plasmids as critical vectors for resistance gene dissemination, with PT_456 representing a high-risk multidrug-resistant lineage.

## Discussion

Shenzhen, a major port city with limited local agriculture and a heavy reliance on external food supplies, provides a unique ecosystem for the importation and local adaptation of MDR *S*. 1,4,[5],12:i:-. Our genomic and epidemiological analysis of 1,908 isolates collected between 2007 and 2023 reveals critical insights into the serovar’s rapid expansion, resistance evolution, and transmission dynamics in this high-risk urban environment.

The proportion of *S*. 1,4,[5],12:i:- among non-typhoidal *Salmonella* isolates surged from 2.27% in 2007 to 24.79% in 2023, consistent with its global rise as a dominant foodborne pathogen and underscoring its establishment as a critical public health threat in Shenzhen [16,51,52]. MLST analysis identified ST34 as the predominant sequence type (97.9%), further confirming its status as a pandemic clone. The emergence of Clade 4 (56.9% prevalence in 2023), a low-diversity lineage (≤ 38 core SNPs), suggests localized adaptation through clonal expansion similar to patterns observed in Europe and North America where ST34 clades have displaced ancestral ST19 due to enhanced environmental fitness and AMR [7].

There has long been debate over the SNP threshold for transmission and outbreaks. Using *Salmonella* as an example, thresholds of 0–10 SNPs have all been applied [42,53]. In fact, the threshold depends not only on the species, but also on the time span of the outbreak and transmission. For instance, a recent study of three *Salmonella* outbreaks showed that the best thresholds were 1, 2, and 7 respectively [54]. In reality, different SNP thresholds will eventually lead to a certain level of false–negative and false–positive results. An early study by Public Health England (PHE) in the UK evaluated the results of different SNP levels from 0 to 10, showing their pros and cons [55]. In our practice, a threshold of 3 SNPs is suitable for tracing bacterial strains [45–48]. Particularly, our analyses indicated approximately 1e-7 substitutions per site per year, or 0.48 SNPs per year. As a result, the 3-SNP threshold can reveal most of the short-term transmission (0–6 years). Based on the above studies, to ensure specificity, this study analysed the effective clusters and number of strains included when the SNP threshold was set between 0 and 20 (Supplementary Table S10 and Supplementary Figure S12), and finally selected a threshold of ≤3 SNPs.

Resistance genes to aminoglycosides, β-lactams, tetracyclines, and sulphonamides were widespread, accounting for > 80% prevalence, reflecting global AMR trends for this serovar [56]. Clade-specific ARGs’ dynamics were observed: Clade 2 showed declining chloramphenicol (*floR*), quinolone (*qnrS1*), and rifamycin (*arr*-3) resistance genes after 2010, aligning with its epidemiological decline. Conversely, Clade 4 exhibited rising resistance genes to chloramphenicol and quinolones after 2013, mirroring its dominance by 2020.

Multiple studies have found a significant association between reduced antibiotic consumption and decreased bacterial resistance, particularly reflected in the resistance changes of third-generation cephalosporins, fluoroquinolones, and aminoglycosides [57–60]. The antimicrobial stewardship campaign launched by the Ministry of Health in 2011 [61], which reduced antibiotic consumption and irrational drug use in Chinese hospitals, may be a key factor contributing to the observed decline in the carriage rates of phenicol, rifamycin, and sulphonamide resistance genes in *S*. 1,4,[5],12:i: – during 2012–2016. Similarly, decreased national antibiotic consumption during the COVID-19 pandemic [62] may explain the reduced isolate counts and ARGs-positive rate in Shenzhen's 2020 isolates. Additionally, antibiotic use in agricultural and livestock industries plays a critical role. The 2017 ban on colistin as a growth promoter by the Ministry of Agriculture [50] may account for the reduced prevalence of the resistance gene *mcr*-1.1. These findings suggest that proper management strategies and policy interventions, along with reducing excessive antibiotic use, could effectively curb the spread of drug-resistant bacteria and need further study.

Plasmid dynamics played a pivotal role in resistance dissemination. The prevalence of IncHI2/2A and pKPC-CAV1321 plasmids declined to 27% in 2023, while Col(pHAD28) prevalence increased to 56.9% in Clade 4, indicating a shift potentially driven by plasmid fitness costs. Col(pHAD28)’s persistence likely stems from its compact genetic load and compatibility with diverse resistance modules, while the rapid expansion of p0111 (60% in Clade 3 by 2023), strongly associated with *dfrA14* and *tet*(A), signals its emerging role in multidrug resistance. KleTy analysis further resolved plasmid-type (PT) specificity: PT_456 emerged as a critical multidrug-resistant hub, carrying aminoglycoside (*aac*, *aph*), ESBL (*bla*_CTX-M-14/65_), and colistin (*mcr*-1.1) resistance genes. PT_6753 universally harboured *sul*2 (100%) and *tet*(B), while PT_464 carried bla_CTX-M-55_ and *qnrS1*. These findings underscore the role of plasmids as reservoirs for resistance genes, with PT_456 and p0111 representing significant threats to public health.

Notably, carbapenem-resistant *bla*_NDM-5_ – harbouring isolates detected in Shenzhen in 2023 all carried plasmids p0111 and IncHI2. In recent years, *bla*_NDM-5_ – carrying *S*. 1,4,[5],12:i: – has been found in clinical patients in Foshan [63] and Zhuhai [64], Guangdong Province, suggesting that *bla*_NDM-5_ can spread widely among Enterobacteriaceae via plasmids IncX3 and IncFII. Studies also indicate that *bla*_NDM-5_ can transfer across bacterial phyla [65]. Moreover, *bla*_NDM_ is frequently detected in non-clinical settings such as surface water, hospital sewage, and wastewater treatment plants [66,67]. These findings highlight Shenzhen’s potential involvement in the Pearl River Delta region’s crisis of carbapenem-resistant Enterobacteriaceae. Overall, these findings underscore the urgent need for antimicrobial stewardship programmes targeting last-line antibiotics and highlight the role of plasmids like IncI1 as high-risk resistance genes vectors.

Transmission network analysis revealed dual epidemic patterns: transient outbreaks (50.6% of clusters lasting < 1 year) linked to acute contamination events and persistent lineages (89.4% of isolates in clusters ≥ 6 months) maintained through reservoirs in livestock or food chains. The median duration of short-term clusters was six months (this value may represent an overestimate because 111/160 clusters [69.4%] lasting < 1 year were excluded from duration calculations due to missing exact dates; the actual median duration might be shorter), indicating localized contamination or transient infection sources. In contrast, cluster 59 and 142 originated in the US and spanned 42 and 61 years respectively. Cluster 59 includes avian strains from 1969 and human strains from 2011. Cluster 142 consists of five human isolates, two food isolates, and one environmental isolate. Notably, two human isolates date back to 1949 and 1953, while the remaining isolates are from 2010. This temporal distribution highlights the serovar's capacity for long-term ecological adaptation and suggests the existence of long-standing hidden transmission sources. Notably, the majority of samples in this study were derived from Shenzhen’s surveillance systems (55.61%), which may reflect surveillance intensity rather than the true prevalence of *S*. 1,4,[5],12:i:-. This sampling approach likely overrepresents clinical cases and specific food sources (e.g. pork products) within Shenzhen compared to environmental or animal reservoirs, potentially skewing temporal and geographic trends.

In Shenzhen, the epidemic was sustained through intra-provincial transmission (0–6 SNPs to Guangdong strains) and international introductions (0–7 SNPs to Canadian isolates). Food handlers played a critical role as transmission bridges, linking contaminated food products to human cases. Of particular significance, four clusters show the coexistence of “food handler-food-patient” elements, confirming the presence of all three components within the same transmission chain. Furthermore, 18 additional clusters directly linked food handlers to patients (≤ 3 SNPs), underscoring the critical role of occupational carriers as key vectors in foodborne outbreaks, and suggesting a plausible “food handler-food-patient” transmission pathway. Similar findings from Guangxi further underscore the role of occupational carriers in outbreaks [25]. While direct epidemiological evidence is absent, tight genomic clustering (≤ 3 SNPs) and the temporal sequence (food handler-food-patient) imply that occupational carriers may bridge foodborne transmission. Future research should integrate traceback investigations to validate this pathway. Phylogenetic analysis linked Shenzhen isolates to outbreaks in the Pearl River Delta, including one cluster involving porcine strains from Guangzhou and three clusters tied to contaminated pork liver in Jieyang.

The dominance of Clade 4 since 2020 illustrates the convergence of adaptive traits: low genetic diversity favouring clonal spread, carriage of Col(pHAD28) plasmids with minimal fitness costs, and rising chloramphenicol (*floR*) and quinolone (*gyrA* p.S83F) resistance genes rates, likely driven by agricultural antibiotic use. The introduction of Clade 4 in 2013 and its rapid rise to a 56.86% prevalence by 2023 suggests strong selective advantages in the Shenzhen environment. This trend aligns with global observations where livestock-associated *Salmonella* strains have adapted and persisted in high-pressure ecological niches [68,69].

Shenzhen’s dependence on imported foods exacerbates cross-border transmission risks, as evidenced by phylogenetic links between Canadian pork-derived isolates and local human cases. The clustering of Shenzhen isolates with strains from other cities in the Pearl River Delta, particularly those originating from Guangzhou and involving porcine sources, highlights the role of regional food supply chains in resistance dissemination.

It is worth noting that, while the inclusion of global genomes strengthens phylogeographic inferences and supports international linkage analyses, the overrepresentation of human clinical isolates compared to food and environmental sources may limit our ability to fully resolve complex transmission pathways. This imbalance could lead to an underestimation of non-human reservoirs and overemphasize direct human-to-human or foodborne links. Future work should prioritize more balanced, source-diverse sampling to improve resolution of One Health transmission dynamics.

To mitigate transmission risks, several interventions are recommended. First, mandatory health screenings and hygiene training for food handlers are essential given their role as occupational carriers. Second, genomic surveillance should prioritize monitoring high-risk and highly transmissible plasmids such as IncHI2/2A, Col(pHAD28), IncI1 and p0111. Third, interprovincial collaboration is necessary to control resistance spread through livestock trade, particularly within the Pearl River Delta's integrated pork industry. Notably, the detection of *bla*_NDM-5_ in three Shenzhen isolates in 2023 demands immediate escalation in carbapenemase surveillance across food and clinical sectors. Finally, it is important to improve the classification and management of antimicrobial drugs, strengthen the regulatory system for antibiotic use, and reduce the misuse of antibiotics.

## Limitations

This study has several limitations. First, the retrospective design limited access to detailed metadata on antibiotic usage in agriculture or clinical settings, hindering causal inferences about resistance selection pressures. Second, the limited number of strains from other countries and regions in the study, along with the focus on Shenzhen, may underrepresent contributions from less surveilled regions in Guangdong and may have affected conclusions about international transmission routes. Third, the absence of longitudinal environmental or animal isolates restricted our ability to fully resolve transmission routes between reservoirs. Finally, while our analysis highlights significant correlations between plasmid types and resistance genes, we acknowledge that these associations do not definitively establish causation. Further functional studies are needed to validate the role of specific plasmids in resistance dissemination.

## Conclusions

This study establishes Shenzhen as a sentinel site for tracking the evolution of multidrug-resistant *S*. 1,4,[5],12:i:- at the human–animal–food interface. The serovar’s rapid clonal expansion, plasmid-driven resistance, and dual transmission patterns underscore the need for integrated One Health interventions in rapidly urbanizing food-import hubs. Strengthening surveillance, managing food supply chains, and monitoring occupational carriers are critical for mitigating public health risks.

## Supplementary Material

Supplementary_Figure_5.jpg

Supplementary_Figure_10.jpg

Supplementary_Figure_3.jpg

Supplementary_Table_S1_S11_revised.xlsx

Supplementary_Figure_4.jpg

Supplementary_Figure_9.jpg

Supplementary_Figure_12.jpg

Supplementary_Figure_1.jpg

Supplementary_Figure_7.jpg

Supplementary_Figure_8.jpg

Supplementary_Figure_2.jpg

Supplementary_Figure_11.jpg

Supplementary_Figure_6.jpg
