# Supplementary figures and images for: Plasmid-driven clonal expansion of multidrug-resistant monophasic Salmonella Typhimurium in a Global Food Trade Hub
Source: Emerg Microbes Infect. 2025 Aug 10;14(1):2542251. doi: 10.1080/22221751.2025.2542251 (PMC12340968; doi:10.1080/22221751.2025.2542251)

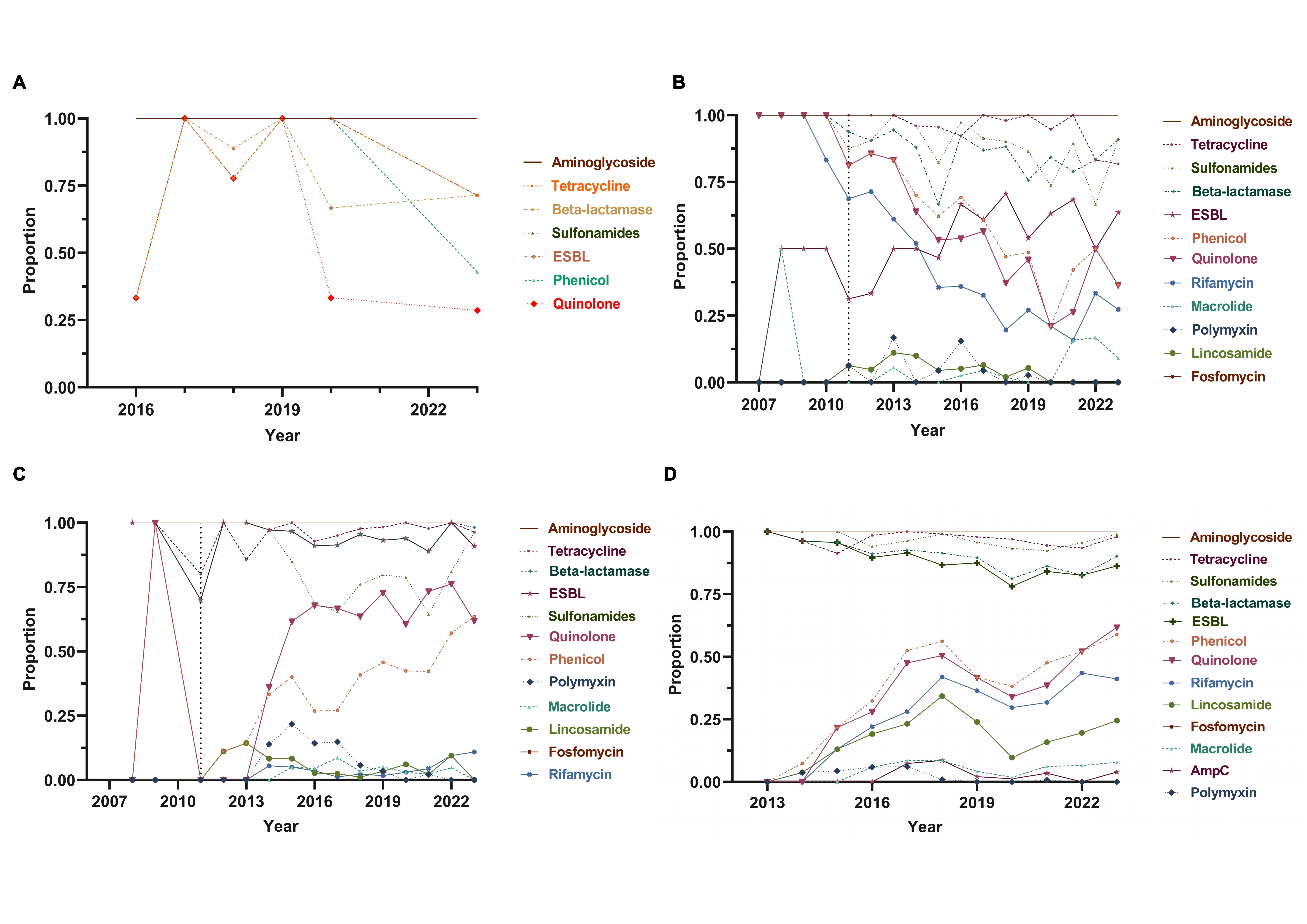

Supplement: Supplementary_Figure_5.jpg [file TEMI_A_2542251_SM5437.jpg]

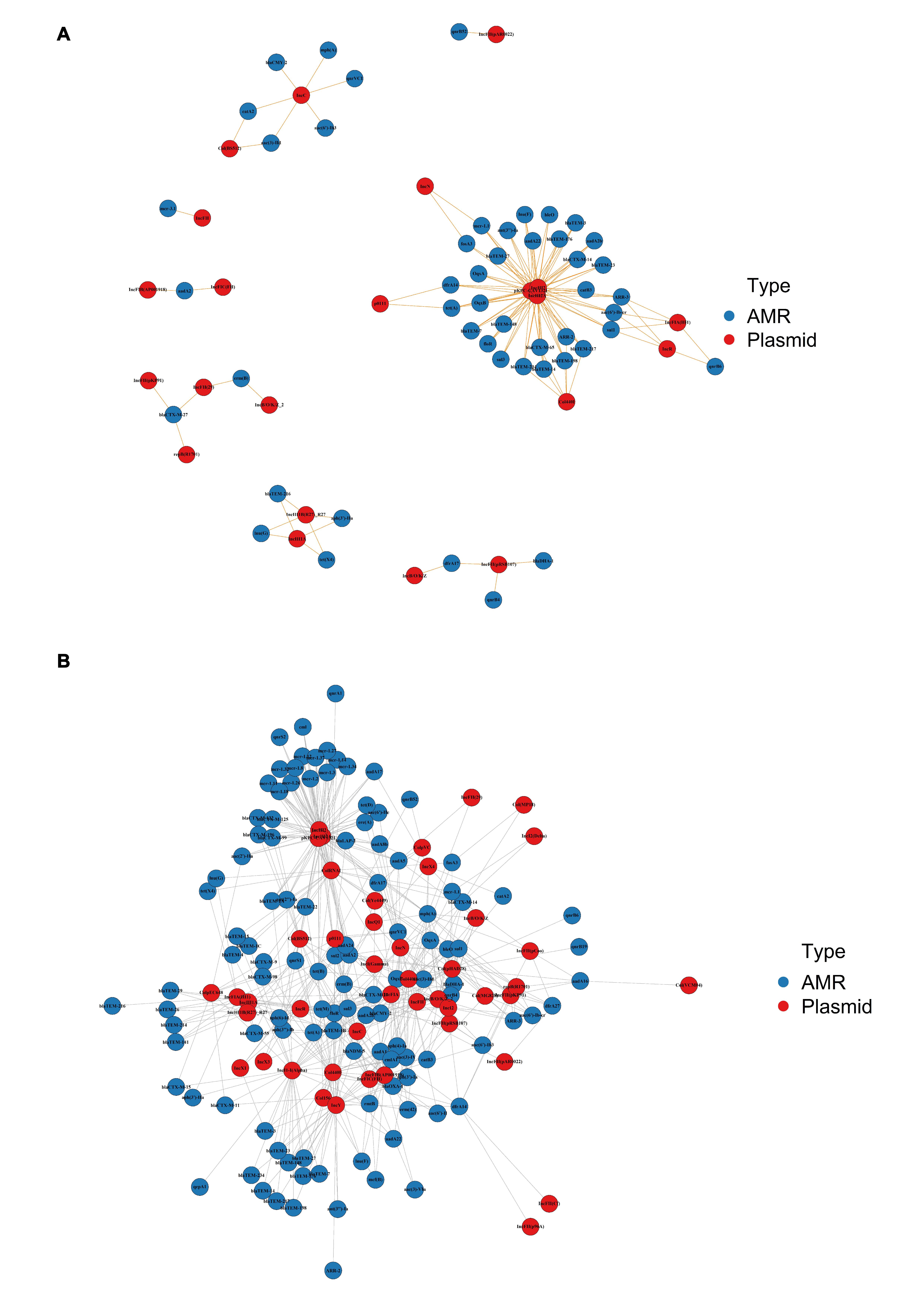

Supplement: Supplementary_Figure_10.jpg [file TEMI_A_2542251_SM5436.jpg]

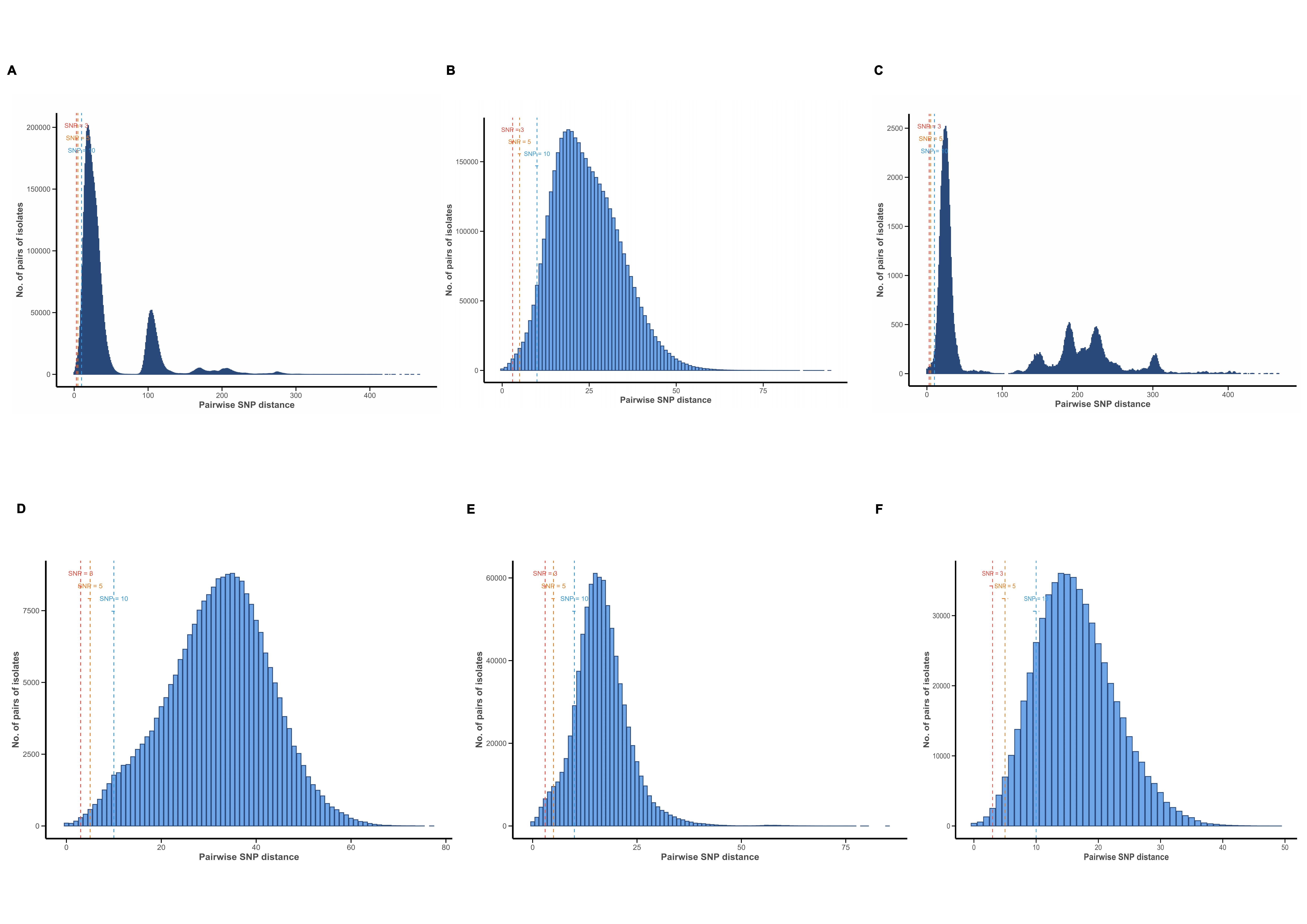

Supplement: Supplementary_Figure_3.jpg [file TEMI_A_2542251_SM5435.jpg]

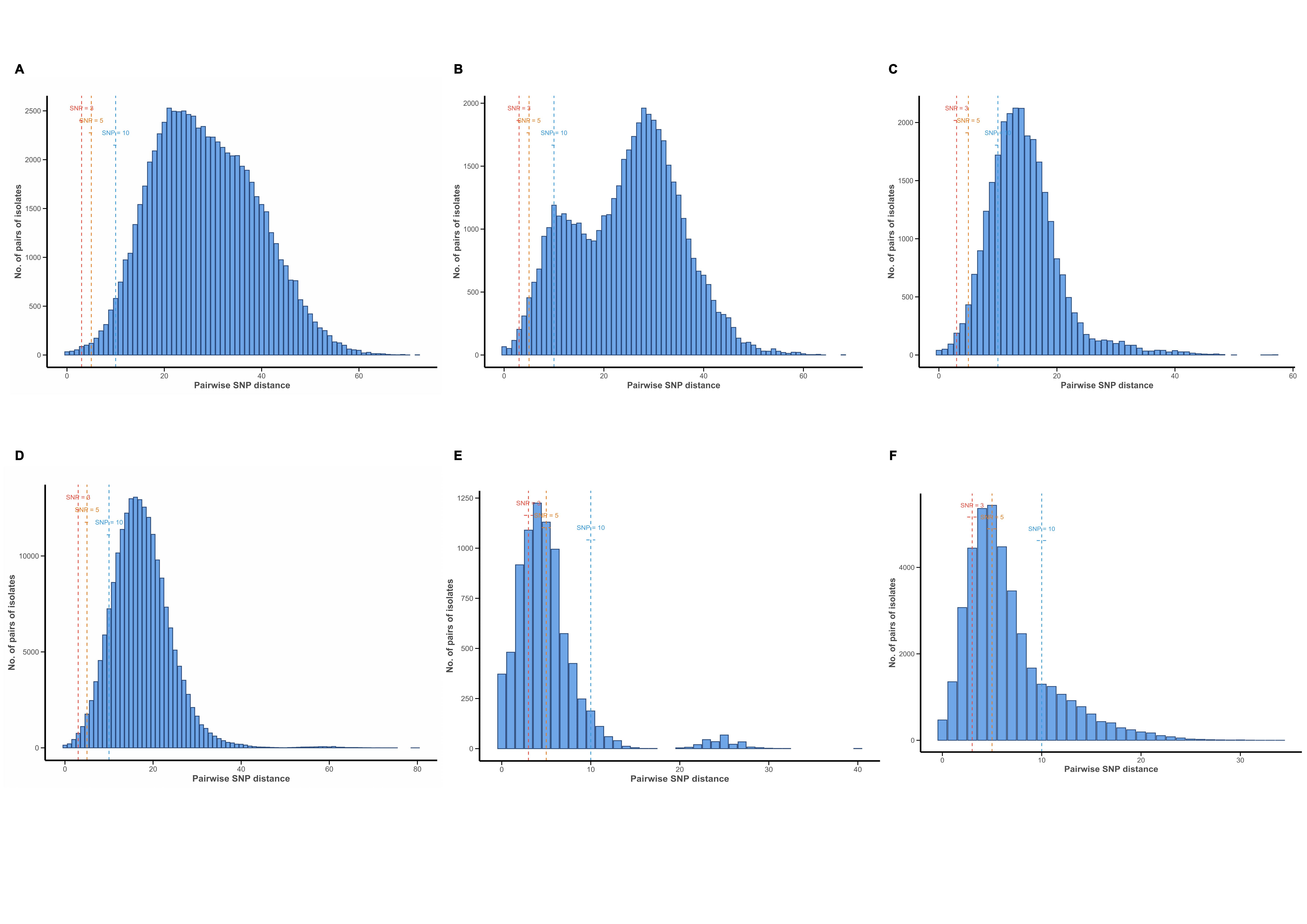

Supplement: Supplementary_Figure_4.jpg [file TEMI_A_2542251_SM5433.jpg]

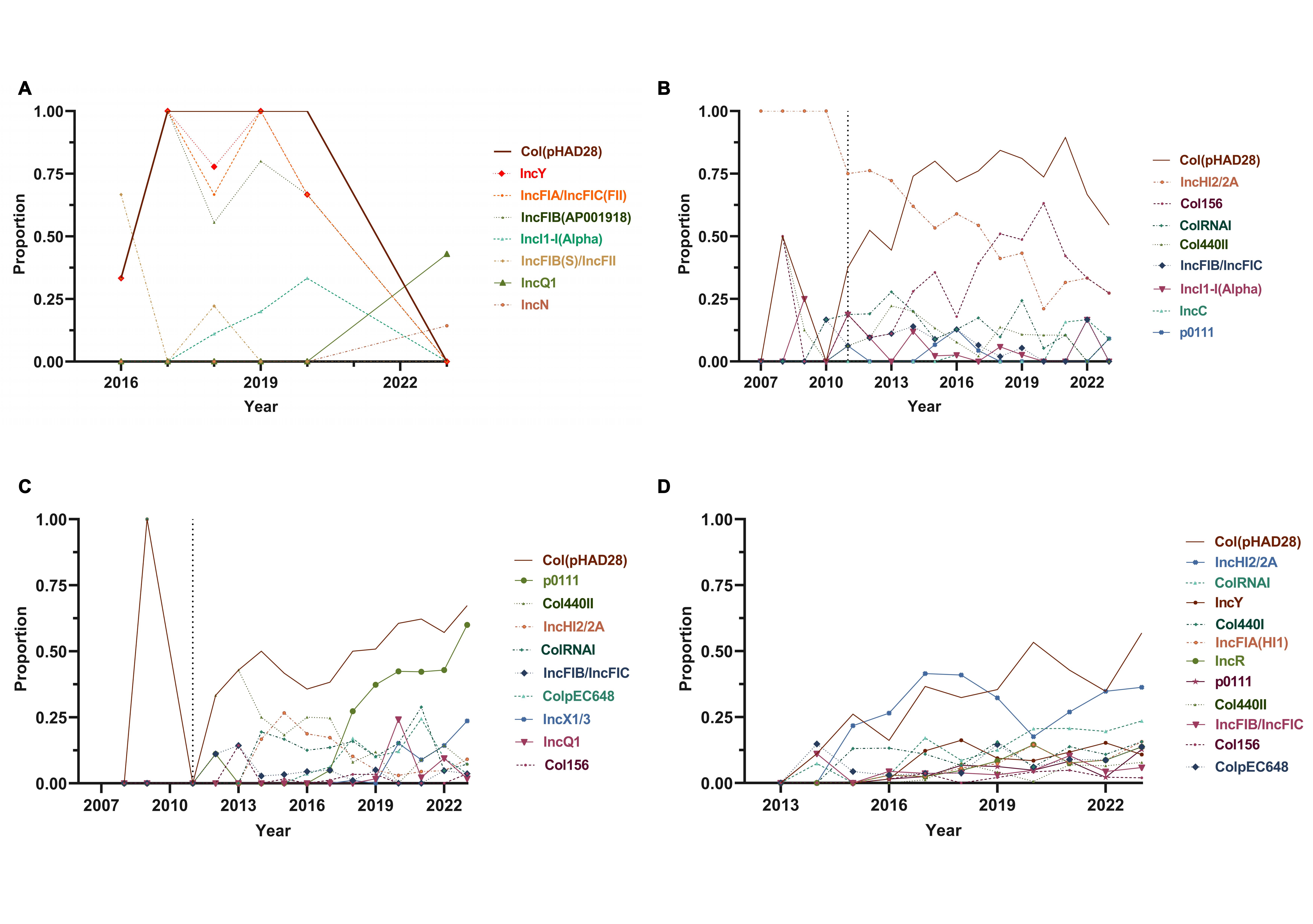

Supplement: Supplementary_Figure_9.jpg [file TEMI_A_2542251_SM5432.jpg]

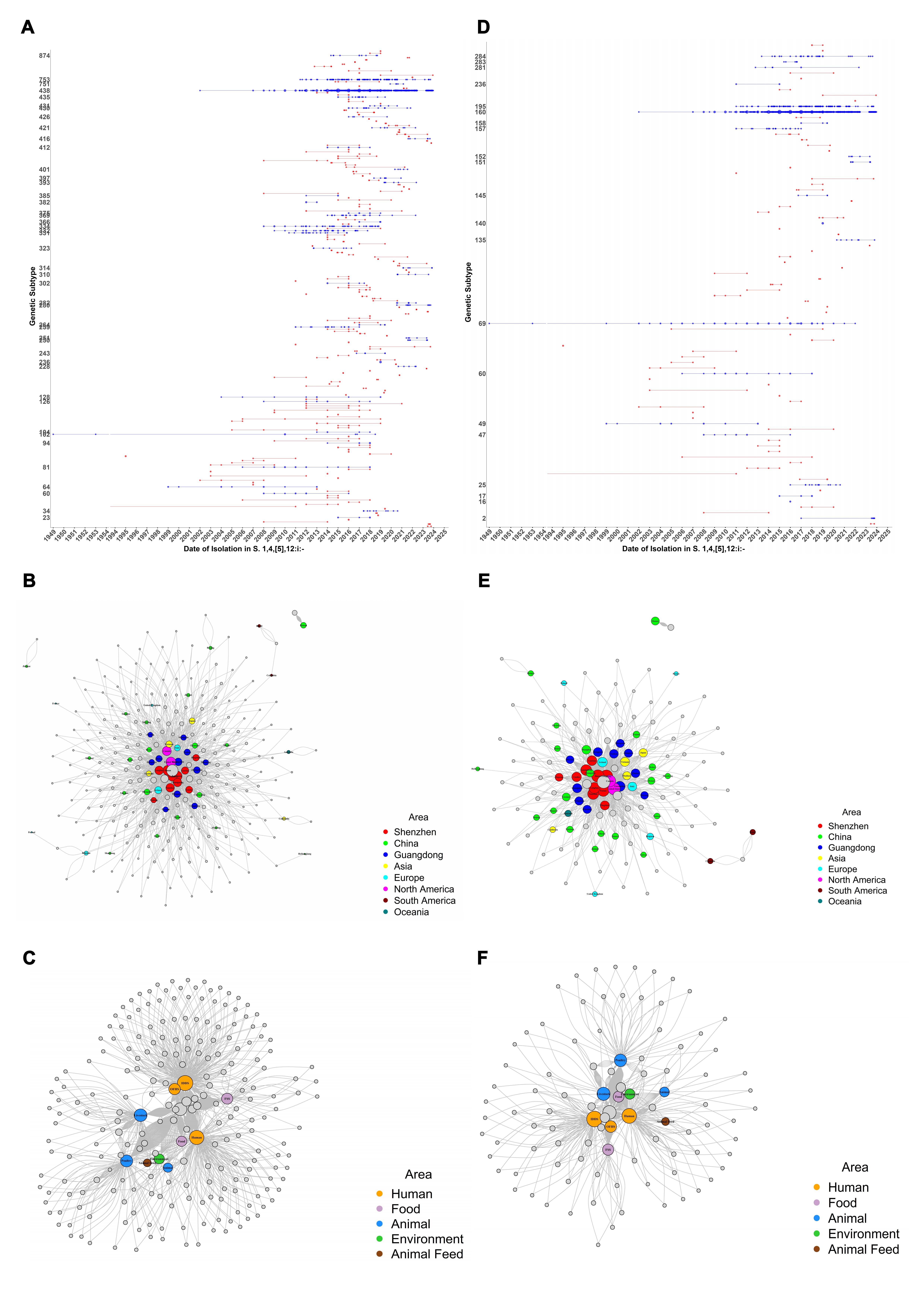

Supplement: Supplementary_Figure_12.jpg [file TEMI_A_2542251_SM5431.jpg]

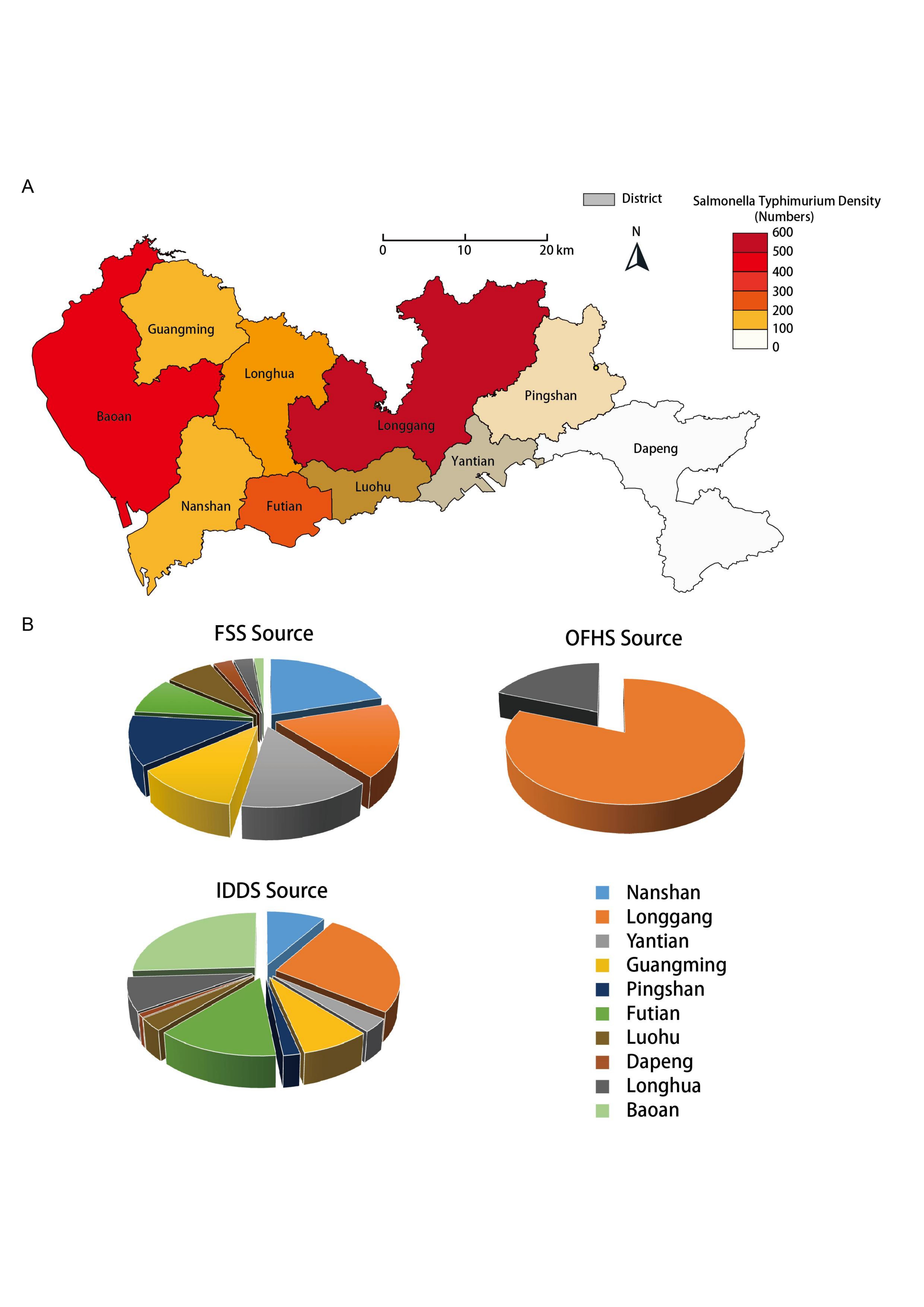

Supplement: Supplementary_Figure_1.jpg [file TEMI_A_2542251_SM5430.jpg]

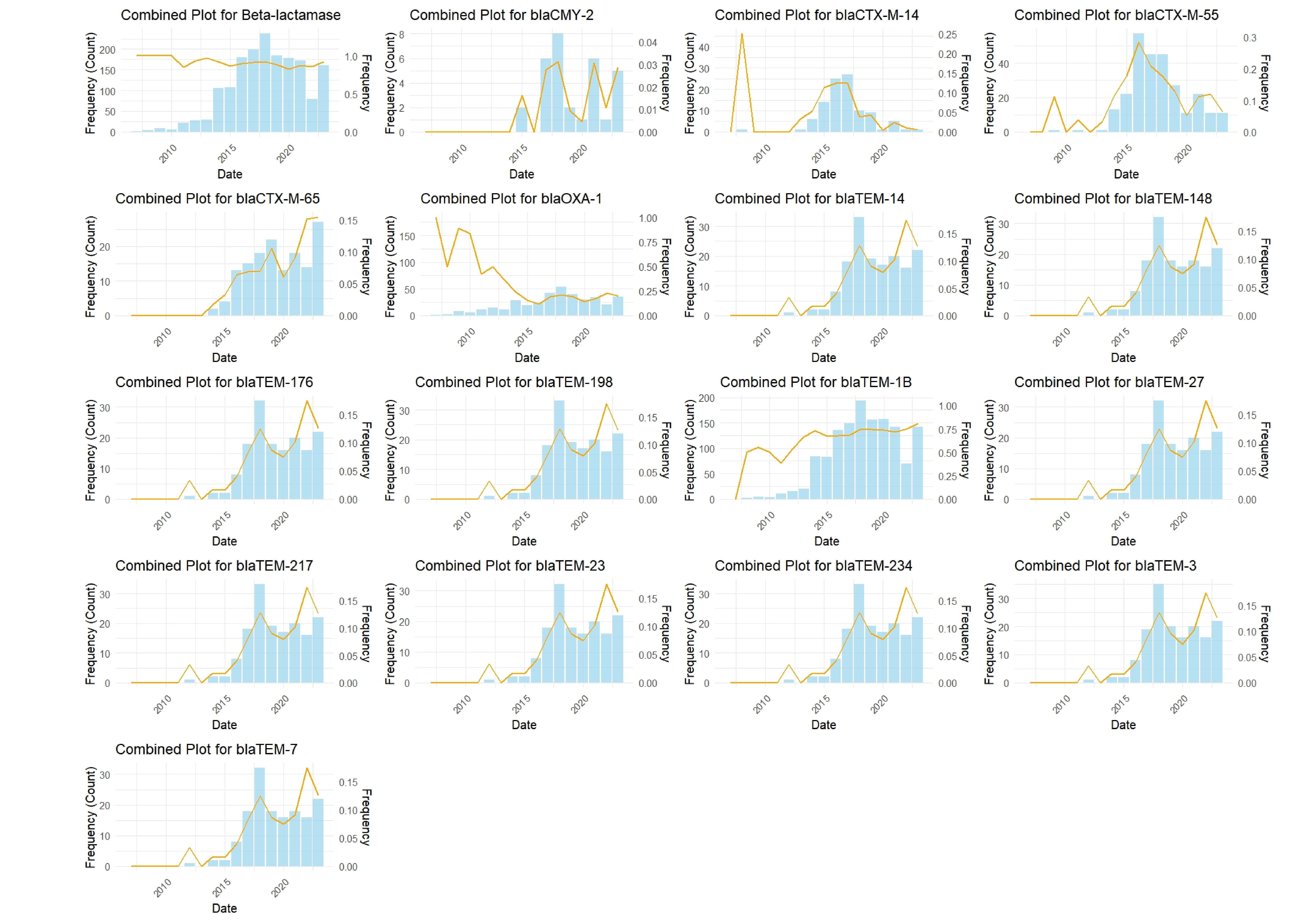

Supplement: Supplementary_Figure_7.jpg [file TEMI_A_2542251_SM5429.jpg]

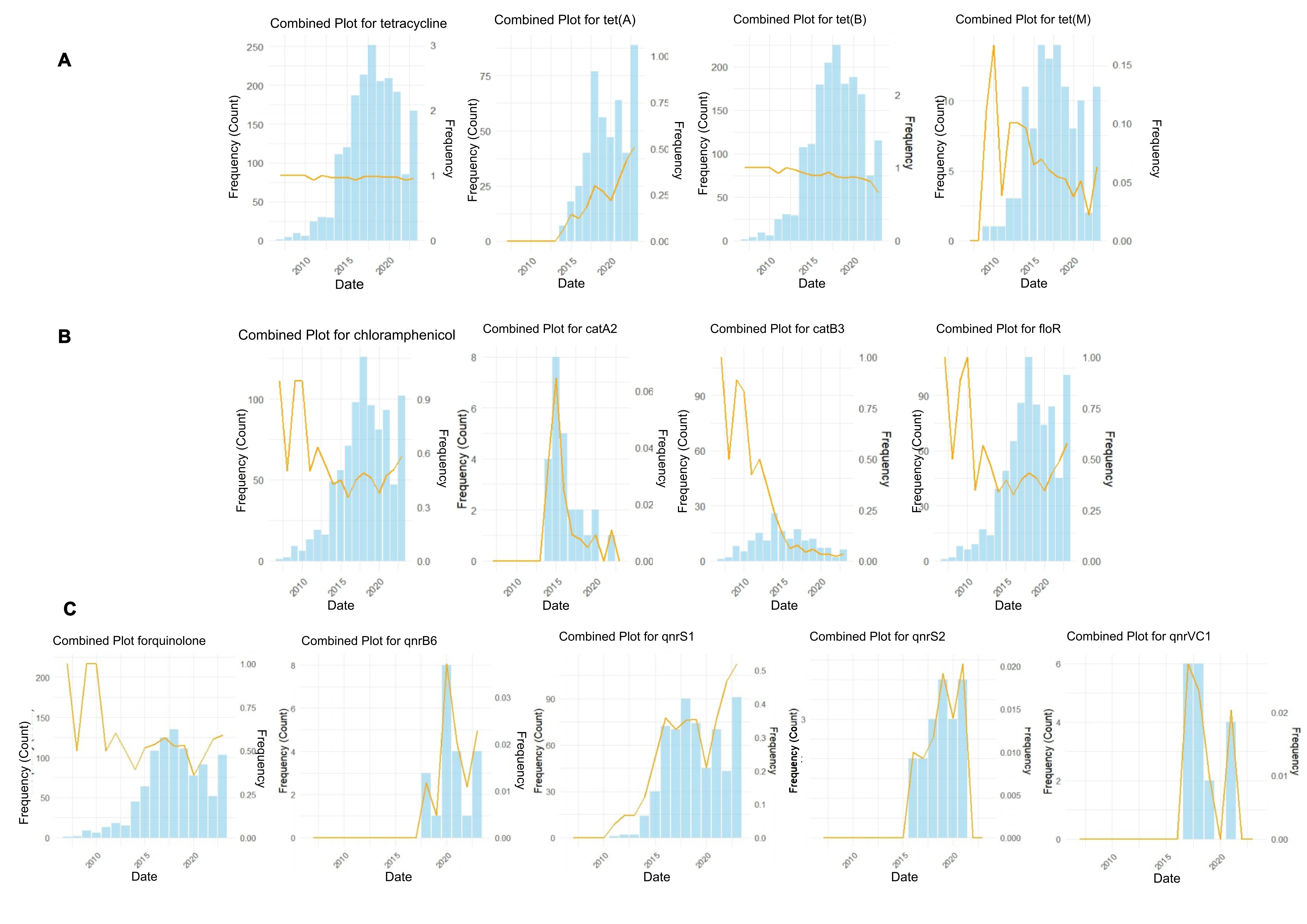

Supplement: Supplementary_Figure_8.jpg [file TEMI_A_2542251_SM5428.jpg]

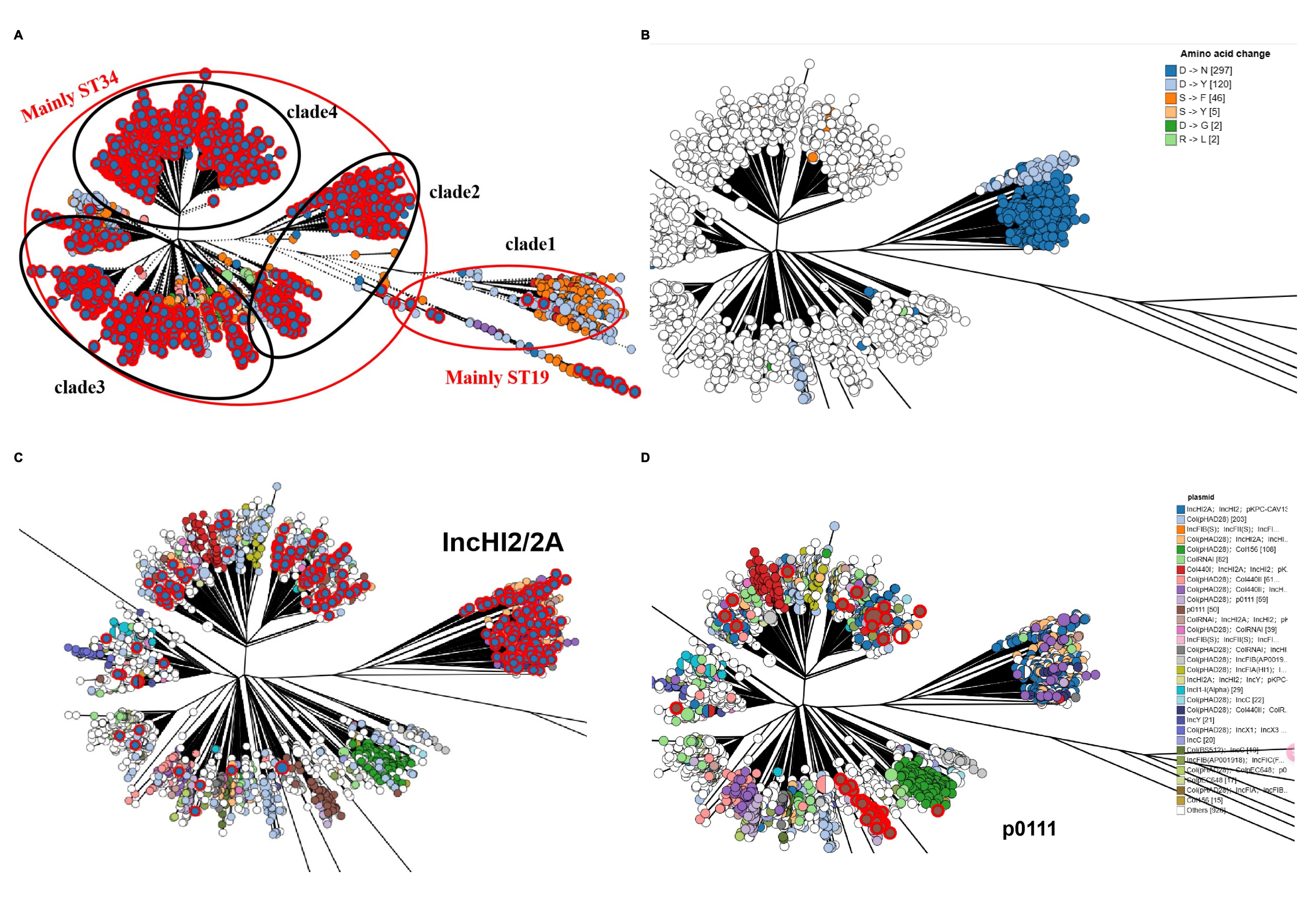

Supplement: Supplementary_Figure_2.jpg [file TEMI_A_2542251_SM5427.jpg]

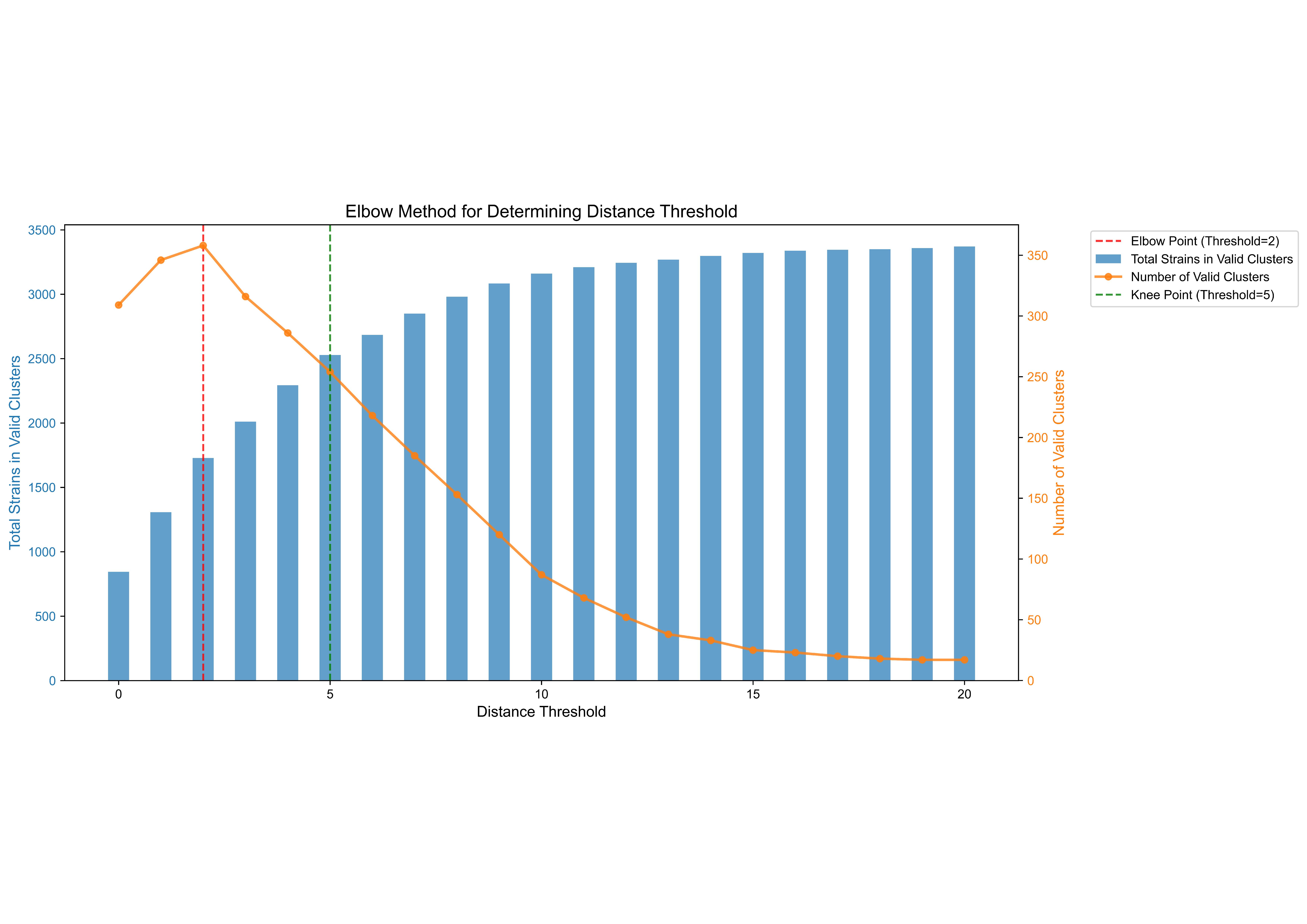

Supplement: Supplementary_Figure_11.jpg [file TEMI_A_2542251_SM5426.jpg]

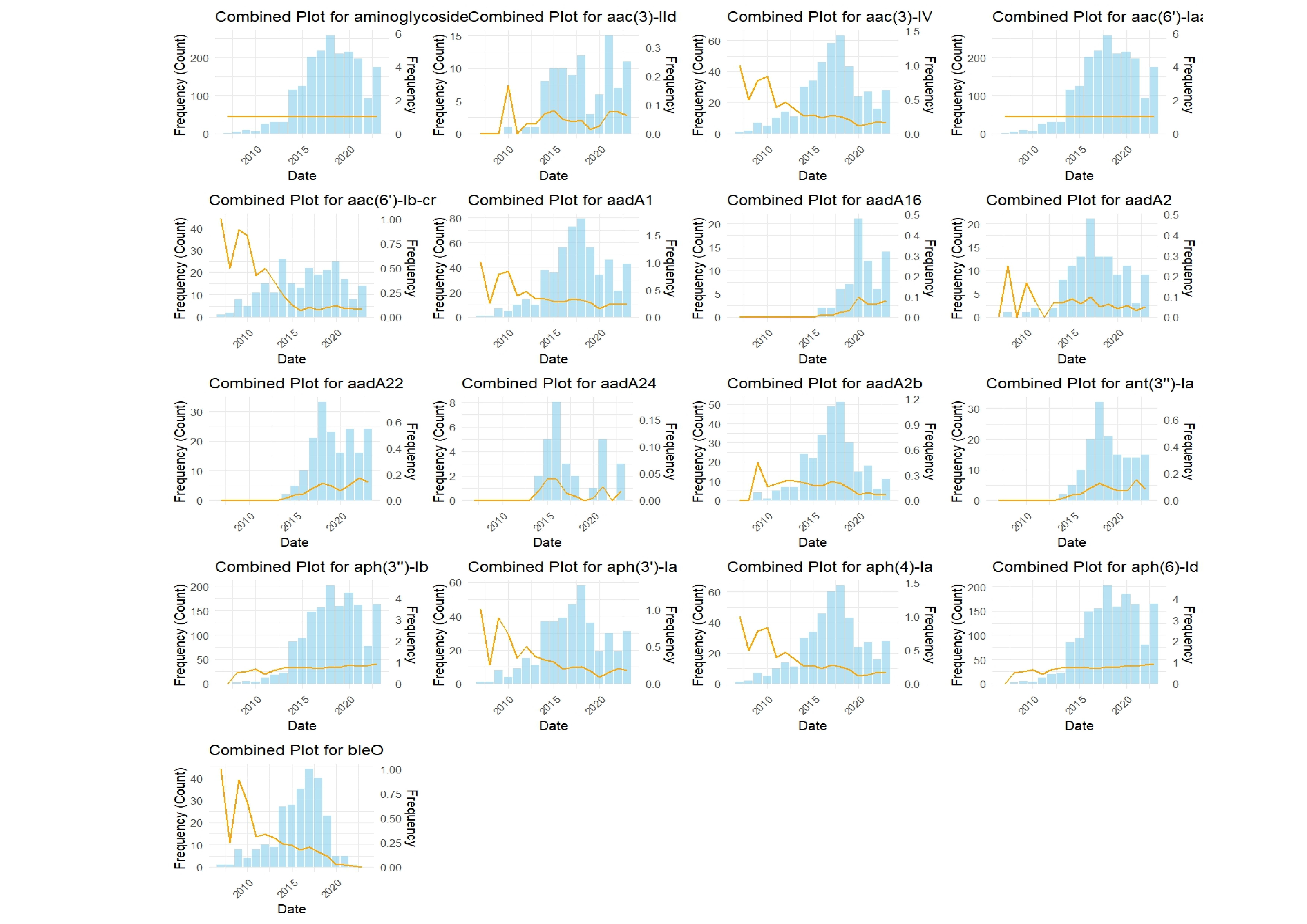

Supplement: Supplementary_Figure_6.jpg [file TEMI_A_2542251_SM5425.jpg]
